# Real-Time Target-Oriented Grasping Framework for Resource-Constrained Robots

**DOI:** 10.3390/s26020645

**Published:** 2026-01-18

**Authors:** Dongxiao Han, Haorong Li, Yuwen Li, Shuai Chen

**Affiliations:** Shanghai Key Laboratory of Intelligent Manufacturing and Robotics, School of Mechatronic Engineering and Automation, Shanghai University, Shanghai 201900, China; handongxiao@shu.edu.cn (D.H.); naonaozhiyu@163.com (H.L.); chenshuaif@shu.edu.cn (S.C.)

**Keywords:** target-oriented grasping, efficient framework, structured pruning, geometry-based grasp correction, resource-constrained platforms

## Abstract

Target-oriented grasping has become increasingly important in household and industrial environments, and deploying such systems on mobile robots is particularly challenging due to limited computational resources. To address these limitations, we present an efficient framework for real-time target-oriented grasping on resource-constrained platforms, supporting both click-based grasping for unknown objects and category-based grasping for known objects. To reduce model complexity while maintaining detection accuracy, YOLOv8 is compressed using a structured pruning method. For grasp pose generation, a pretrained GR-ConvNetv2 predicts candidate grasps, which are restricted to the target object using masks generated by MobileSAMv2. A geometry-based correction module then adjusts the position, angle, and width of the initial grasp poses to improve grasp accuracy. Finally, extensive experiments were carried out on the Cornell and Jacquard datasets, as well as in real-world single-object, cluttered, and stacked scenarios. The proposed framework achieves grasp success rates of 98.8% on the Cornell dataset and 95.8% on the Jacquard dataset, with over 90% success in real-world single-object and cluttered settings, while maintaining real-time performance of 67 ms and 75 ms per frame in the click-based and category-specified modes, respectively. These experiments demonstrate that the proposed framework achieves high grasping accuracy and robust performance, with a efficient design that enables deployment on mobile and resource-constrained robots.

## 1. Introduction

Robotic systems have seen rapid progress and have been applied across diverse domains, including autonomous aerial and ground platforms, service and logistics robots, and underwater and soft robotic systems [1,2,3,4]. In many of these applications, robotic grasping is a fundamental capability for robots to perceive, interact with, and manipulate objects in real-world environments. In practice, robots are often required to perform target-oriented grasping, in which a specified object is grasped according to user instructions. This capability is critical in domains such as industrial assembly, household assistance, and teleoperation, where robots must efficiently identify, localize, and manipulate objects for tasks such as tool delivery, provision of daily necessities, or pick-and-place operations [5,6,7,8].

Traditional grasping approaches typically rely on geometric modeling or analytical computation [9,10,11], which require precise object representations and exhibit limited generalization. These have affected their applicability in complex and dynamically changing environments. With the rapid development of deep learning, data-driven grasping methods [12,13,14,15] have become widely adopted, as they predict grasp poses directly from visual input and achieve higher accuracy and better generalization across diverse objects and scenes. However, most existing methods focus solely on grasp feasibility and cannot grasp a user-specified target object. Some of these studies have also integrated object detection and segmentation modules to identify target objects and estimate grasp poses [16,17,18]. Although these grasping networks are lightweight and suitable for deployment on consumer-grade or embedded robotic hardware, their dependence on predefined object categories limits their generalization capability, particularly when encountering unseen objects in real-world settings.

Recently, language-driven target-oriented grasping [5,19,20] has attracted increasing research interest. Natural language understanding enables robots to execute object-specific grasping based on semantic instructions, which can enhance flexibility and task understanding in human–robot interaction. Despite being more intuitive than visual-only cues, free-form language instructions introduce challenges in cross-modal reasoning and the precise inference of user intent [21]. Moreover, existing language-driven approaches typically rely on large-scale language models (LLMs), require considerable computational and memory resources, and exhibit high inference latency, which can limit their deployment on resource-constrained household and industrial robots [22,23].

This work is motivated by the growing need for target-oriented grasping methods that combine semantic understanding with computational efficiency on consumer-level or edge devices, so that the robots can accurately identify and grasp specified objects without relying on large-scale language models. To overcome the limitations of existing approaches, including limited semantic reasoning, poor generalization to novel objects, and insufficient real-time performance, this work proposes a lightweight and deployable grasping framework, which integrates object detection and segmentation to support the precise grasping of known objects and allows rapid interaction with previously unseen objects.

The main contributions of this work are summarized as follows:

1. A target-oriented grasping framework with intuitive interaction is presented, supporting click-based selection for unknown objects and category-based specification for known objects.
2. A lightweight vision-based grasping pipeline is developed, where a pruned YOLOv8 localizes target objects in category-based mode and GR-ConvNetv2 with MobileSAMv2 generates grasping proposals. A geometry-based correction module further refines the grasping position, angle, and width to improve the accuracy.
3. Extensive experiments on benchmark datasets and real-world scenarios, including single-object, cluttered, and stacked settings, show that the proposed approach is well suited for deployment on resource-constrained mobile robots.

## 2. Related Works

This section reviews the related work on vision-based robotic grasping and target-oriented grasping. First, representative vision-based grasping methods are summarized, focusing on grasp representations, learning-based grasp pose prediction, and real-time performance. Next, target-oriented grasping approaches are reviewed, which incorporate object-level semantics or high-level instructions to enable the grasping of specified targets. In particular, recent advances leverage large language models (LLMs), vision–language models (VLMs), and vision–language–action (VLA) models to bridge semantic understanding and grasp execution through multimodal reasoning.

### 2.1. Vision-Based Robotic Grasping

Early convolutional-based methods for robotic grasping focused on designing efficient grasp representations and learning-based predictors. A grasp rectangle representation was proposed to encode a seven-dimensional gripper configuration in the image plane, combined with a two-step learning algorithm that generates candidate grasps and ranks them for accurate estimation from RGB-D images [12]. Building on this grasp-rectangle representation, a single-stage CNN regression framework was developed to directly predict grasp rectangles from RGB-D images for real-time inference without a sliding-window search [13]. To improve the data efficiency and robustness, a large-scale self-supervised grasping dataset was collected via robotic trial-and-error, which enables a CNN to predict the grasp success probabilities directly from raw image patches and generalize to novel objects [24]. In real-world multi-object scenes, a single shot classification-based network predicts multiple grasp candidates per object for efficient multi-grasp detection in clutter [25]. A real-time generative grasp-synthesis method was proposed, in which a Generative Grasping Convolutional Neural Network (GG CNN) predicts the grasp quality, angle, and width at every pixel from a depth image, which allows closed-loop grasping at rates up to 50 Hz [14]. A different grasp parameterization using a new oriented diameter circle representation was later proposed to regress the grasp center, orientation, and width with intuitive geometric semantics [26]. To generate physically plausible antipodal grasps, a Generative Residual Convolutional Neural Network (GR-ConvNetv2) was developed, capable of synthesizing grasp configurations from n channel inputs in real time [27]. More recently, a transformer-based architecture (TF Grasp) was deployed to model both local and cross-window attention, which captures long-range dependencies and context for more robust grasp detection in cluttered scenes [28]. However, these approaches primarily predict grasp poses in real time without incorporating semantic information, limiting their ability to target specific objects.

### 2.2. Target-Oriented Grasping

Target-oriented grasping extends traditional robotic grasping beyond generic pose prediction to focus on task-specific and target-oriented grasping. Approaches that integrate object detection with grasp estimation have been proposed to overcome the limitations of purely vision-based grasping. For example, a parallel YOLO–GG framework combining object detection and grasp pose prediction was proposed to allow collaborative robots to identify and manipulate specific targets [18]. An end-to-end, computationally lightweight vision-to-grasp system was also developed for mobile manipulators to handle daily objects, by unifying the object detection, pose estimation, and grasp point prediction to achieve real-world pick-and-pack performance on embedded hardware [29]. In addition, several other detection-and-grasping methods have been proposed in the literature [30,31,32].

Recent advances have utilized LLMs, VLMs, and VLA models to enable reasoning over both object attributes and specific parts described in natural language commands, to enhance the robot’s ability to perform context-aware grasping. For instance, reasoning grasping integrates a multimodal LLM with vision-based grasp detection to produce object- and part-level grasps from natural instructions while considering scene context [19]. Similarly, GraspGPT exploits semantic knowledge from LLMs to predict task-oriented grasps for zero-shot generalization to novel objects [5]. Open-vocabulary frameworks, such as OVGrasp, align textual embeddings with visual features to allow the grasping of unseen categories in cluttered scenes [20], and other studies have explored LLM-based approaches for target-oriented grasping [33,34]. In addition to reasoning over object semantics and visual features, ambiguities in natural language commands and challenges in occluded or cluttered scenes have also been addressed in the literature. For example, a command-driven semantic grasp architecture was developed in which a visual-attribute recognition module with pixel-attention was combined with a modified grasp-map-estimation network to map natural-language task commands to the grasp quality, angle, and width through semantic attributes [35]. Recent VLA models further unify vision, language, and action by augmenting pretrained VLMs or LLMs with action prediction modules. For example, VLA-Grasp uses a prompted LLM for task inference together with multimodal encoders and cross-attention fusion to integrate vision, language, and action for task-constrained grasp prediction [36]. DexGraspVLA leverages a pretrained VLM as a high-level task planner and combines it with a diffusion-based controller to realize general dexterous grasping over diverse objects and scenarios [37].

To facilitate comparison, Table 1 summarizes the representative target-oriented grasping methods discussed above in terms of the work category, applied methods, key strengths, limitations, inference time, and hardware requirements.

Overall, while the detection-and-grasp methods demonstrate strong performance in fast and precise grasp estimation for known objects, they are constrained by pretrained object categories. LLM-, VLM-, and VLA-based methods incorporate semantic reasoning and language-conditioned manipulation but face challenges in accurately mapping high-level instructions to graspable regions and achieving real-time inference on resource-constrained robotic platforms. Compared to these existing approaches, the method proposed in this work supports two interactive modes that handle both known and unknown objects, performs grasp pose correction to cope with complex scenarios such as stacked arrangements, and achieves efficient inference on consumer-level platforms without requiring the high computational power needed by large models.

## 3. Method

This section presents the proposed efficient framework for real-time target-oriented robotic grasping. We first provide an overview of the system architecture, highlighting how grasp pose prediction, user instruction, object detection, and segmentation are integrated into a unified pipeline. We then describe the pruned YOLOv8-based object detection model and the structured pruning strategy adopted to reduce the computational cost while preserving the detection accuracy. Next, the grasp pose generation process is detailed, including segmentation-guided grasp selection using MobileSAMv2 and a geometry-based correction algorithm for refining the grasp pose, angle, and width. Finally, the grasping datasets, evaluation metrics, and quantitative results are presented.

### 3.1. System Overview

**Figure 1:**
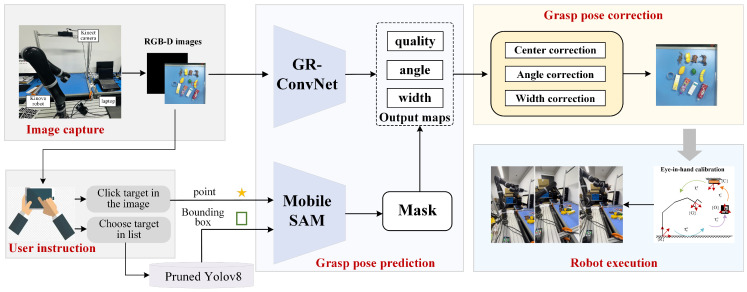
Overview of the proposed target-oriented grasping system.

An overview of the proposed target-oriented grasping system is shown in Figure 1. The system is designed with a focus on balancing the grasp accuracy, interaction flexibility, and real-time performance on resource-constrained platforms. RGB and depth images captured by the camera are first processed by a pretrained GR-ConvNetv2 [27] to produce the initial grasp proposals (the red rectangle in the RGB image), represented by the grasp quality, angle, and width maps. Meanwhile, the RGB image is displayed on the user interface to facilitate user instruction. Two complementary modes of user instruction are supported to enhance the interaction flexibility: click-based selection for unknown objects and category-based specification for known objects. In the click-based mode, a user-selected pixel is forwarded to the MobileSAMv2 [38], whereas in the category-based mode, a pruned YOLOv8 model detects the target object and provides its bounding box. MobileSAMv2 then generates a binary mask based on the selected point or bounding box, constraining the candidate region on the grasp quality map and ensuring that the subsequent grasp pose correction is both accurate and computationally efficient. A geometry-based correction algorithm is applied to further refine the position, angle, and width of the selected grasp pose, enhancing the grasp reliability while maintaining low computational overhead. The corrected pose (the blue rectangle in the RGB image) is finally transmitted to the robotic arm controller to execute the target-oriented grasp. Details of the pruned YOLOv8 detection model and grasp pose generation are provided in the following sections.

### 3.2. Object Detection

#### 3.2.1. YOLOv8 Model

Object detection algorithms are typically categorized into two-stage detectors and single-stage detectors. Among single-stage detectors, YOLO has demonstrated strong performance in real-time applications due to its end-to-end design and high inference speed. Considering both accuracy and real-time requirements, YOLOv8 [39] is adopted as the backbone for object detection due to its maturity, stable performance, and relatively low computational overhead compared to higher-version YOLO variants with increased architectural complexity.

**Figure 2:**
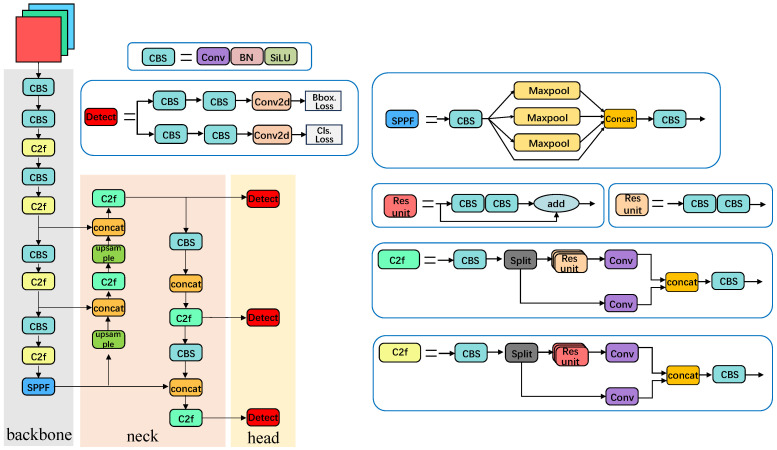
YOLOv8 architecture.

YOLOv8 extends the YOLOv5 architecture through a series of structural optimizations aimed at improving the feature extraction and detection efficiency, as shown in Figure 2. In the backbone, the lightweight C2f module replaces the C3 module from YOLOv5, enabling more effective feature reuse and gradient propagation, while the SPPF module is retained to enhance multi-scale feature aggregation. In the neck, YOLOv8 maintains the Feature Pyramid Network (FPN) and Path Aggregation Network (PAN) structures to fuse multi-scale features, thereby strengthening the model’s capability to detect objects of different scales. The decoupled detection head separates classification and regression, optimized by class and bounding-box losses, improving the accuracy and convergence. Further architectural details can be found in [39].

#### 3.2.2. Structured Pruning

Although YOLOv8 achieves high accuracy in object detection, its large number of parameters limits deployment on resource-constrained devices, which makes model compression necessary. Among common compression strategies, including quantization [40,41], knowledge distillation [42,43], and architecture redesign [44,45,46], structured pruning provides an effective approach by removing redundant computations while preserving the overall network structure. Meanwhile, pruning ratios can be adjusted flexibly, based on the desired accuracy and inference speed. In this work, structured pruning is applied to YOLOv8 using the Dependency Graph (DepGraph) method [47]. DepGraph is a model-agnostic structured pruning framework that automatically identifies parameter dependencies across various network architectures, including convolutional, recurrent, graph neural networks, and vision transformers, making the pruning strategy transferable to other detectors. For the YOLOv8 backbone used in this work, the pruning configuration and layer selection are specifically adapted, considering its backbone, neck design, and decoupled detection head, to ensure pruning stability and performance.

The pruning procedure begins with constructing a dependency graph to identify parameter groups that must be pruned simultaneously. To do this, each network module, such as convolution, batch normalization, or residual connection, is represented as an operation unit $f_{i}$, with input and output denoted as $f_{i}^{-}$ and $f_{i}^{+}$, respectively. The network can be expressed as $F=\{f_{1}^{-},f_{1}^{+},f_{2}^{-},f_{2}^{+},\ldots ,f_{L}^{-},f_{L}^{+}\}$. This representation facilitates easier dependency modeling and allows distinct pruning schemes for the same layer. By redefining the neural network as Equation (1), two primary types of dependencies can be identified: inter-layer dependencies and intra-layer dependencies.

(1) $$\left(f_{1}^{-},f_{1}^{+}\right)↔\left(f_{2}^{-},f_{2}^{+}\right)\ldots ↔\left(f_{L}^{-},f_{L}^{+}\right),$$

where ↔ represents the connectivity between two adjacent layers.

Inter-layer dependencies occur between connected layers, i.e.,

(2) $$f_{i}^{-}\Leftrightarrow f_{j}^{+}\;\;\mathrm{if}\;f_{i}^{-}↔f_{j}^{+}.$$

Intra-layer dependencies arise when a layer’s input and output require a consistent pruning scheme, such as batch normalization layers or residual channels, which is expressed as

(3) $$f_{i}^{-}\Leftrightarrow f_{i}^{+}\;\;\mathrm{if}\;sch\left(f_{i}^{-}\right)=sch\left(f_{i}^{+}\right).$$

Based on these dependencies, a dependency matrix *D* can be constructed, as follows:

(4) $$D\left(f_{i}^{-},f_{j}^{+}\right)=\{\begin{array}{cc} 1 & \mathrm{if}\;f_{j}^{-}↔f_{i}^{+}\mathrm{or}\left(i=j\;\mathrm{and}\;sch\left(f_{i}^{-}\right)=sch\left(f_{i}^{+}\right)\right)\\ 0 & \mathrm{otherwise} \end{array},$$

where sch(·)=sch(·) denotes that the corresponding feature maps share identical pruning schemes. This symmetric matrix captures both inter- and intra-layer relationships, allowing parameters with dependencies to be grouped for synchronous pruning.

To enforce consistent sparsity within each group, group sparsity regularization is applied during training using an $L_{2}$ regularization term:

(5) $$R\left(g,k\right)=\sum_{k=1}^{K}\gamma_{k}I_{g,k}=\sum_{k=1}^{K}\sum_{w\in g}\gamma_{k}{∥w\left[k\right]∥}_{2}^{2},$$

where $I_{g,k}=\sum_{w\in g}{∥w\left[k\right]∥}_{2}^{2}$ represents the importance of the $k^{th}$ prunable dimension in group *g*. $\gamma_{k}$ is the shrinkage strength, computed as

(6) $$\gamma_{k}=2^{\alpha \left(I_{g}^{\max}-I_{g,k}\right)/\left(I_{g}^{\max}-I_{g}^{\min}\right)},$$

where α=4 is used in this work. After sparse training, a relative score is computed to rank parameter importance, and parameters with low scores are subsequently removed:

(7) $${\hat{I}}_{g,k}=N\cdot I_{g,k}/\sum \mathrm{Top}N\left(I_{g}\right).$$

A detailed description of the pruning operations and their results is given in Section 4.2, and further theoretical insights on pruning can be found in [47].

### 3.3. Grasp Pose Generation

#### 3.3.1. Grasp Pose Prediction

In this work, the GR-ConvNetv2 model is employed to predict grasping poses. It has been demonstrated that this model can achieve an inference speed of about 20 ms per image and demonstrates robust generalization to unseen objects in cluttered scenes on a system with an Intel Core i7-7800X CPU and an NVIDIA GeForce GTX 1080 Ti GPU [27].

**Figure 3:**
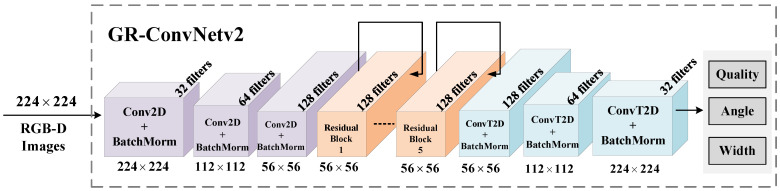
GR-ConvNetv2 model.

As shown in Figure 3, GR-ConvNetv2 takes RGB-D images of size 224 × 224 and extracts features through convolutional and residual layers, reducing the resolution to 56 × 56. Three transpose convolutional layers then upsample the features to generate grasp quality, angle, and width maps aligned with the input. The predicted grasp pose can be formally represented as

(8) $$G=\left(Q,\theta ,W\right)\in R^{3\times h\times w},$$

where *Q*, θ, and *W* denote the pixel-wise grasp quality, angle, and gripper width, respectively, and h×w corresponds to the input image resolution.

To constrain the grasp poses to the target object and reduce the impact of incorrect high-quality predictions in surrounding regions, a segmentation-guided masking strategy is adopted. Upon receiving the user’s instruction, which could either be a click corresponding to a point on the object or a category specifying the object’s bounding box, a segmentation prompt is generated accordingly for MobileSAMv2 to produce a binary mask of the target object. The generated binary mask *M* is then applied to the grasp quality map *Q* to refine the predicted grasp poses:

(9) $$Q^{\prime }=Q\cdot M.$$

Among them, the candidate grasp pose *g* of the target object is selected at the pixel (x,y) with the maximum quality value in $Q^{\prime }$, with the corresponding angle and width defining the gripper configuration:

(10) g=(x,y,θ,W).

**Figure 4:**
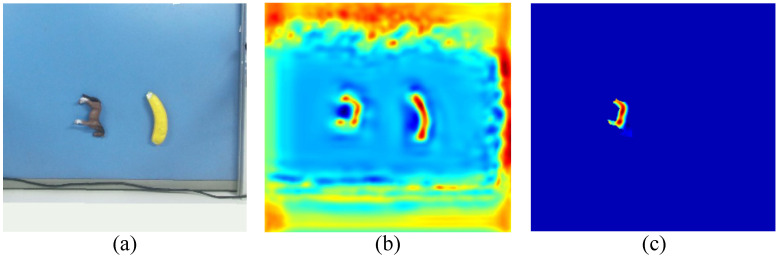
Segmentation-guided grasp quality masking. (**a**) Input RGB image. (**b**) grasp quality map before masking. (**c**) Masked target-object quality map. The color scale indicates grasp quality, where warmer colors represent higher grasp confidence.

Figure 4 illustrates the effect of the segmentation-guided masking strategy. The input RGB image is shown in Figure 4a, and the grasp quality map before masking is shown in Figure 4b, where high scores can be observed on background regions. After applying the segmentation mask, the refined quality map in Figure 4c highlights high scores only on the target object, demonstrating that the proposed strategy effectively constrains grasp predictions to the intended object and prevents erroneous grasps on non-target areas.

#### 3.3.2. Grasp Pose Correction

**Figure 5:**
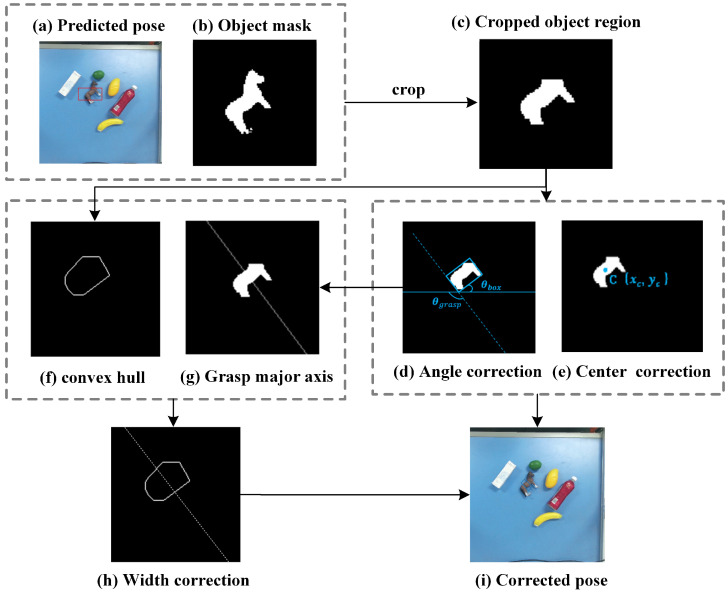
Correction process for grasp poses.

As shown in Figure 5a, the grasp pose (the red rectangle) predicted by GR-ConvNetv2 and MobileSAMv2 may exhibit center offsets, angle errors, or inappropriate gripper widths, which can cause execution failures such as collisions with other objects in cluttered scenes. To improve the grasp performance, a geometry-based correction algorithm is applied to adjust the center, angle, and width of the grasp pose, resulting in a corrected grasp pose (the blue rectangle).

The overall geometry-based grasp correction procedure is summarized in Algorithm 1. To correct the predicted grasps, the predicted grasp pose (see Figure 5a) and the corresponding object mask (see Figure 5b) are used to crop the object region within the rectangle (see Figure 5c). We first correct the center by computing the centroid $\left(x^{\prime },y^{\prime }\right)$ of the object region (see Figure 5e) using image moments:

(11) $$x^{\prime }=\frac{M_{10}}{M_{00}},\;y^{\prime }=\frac{M_{01}}{M_{00}},$$

where the moments are defined by $M_{00}=\Sigma_{u}\Sigma_{v}I\left(u,v\right)$, $M_{10}=$$\sum_{u}\sum_{v}uI\left(u,v\right),\;\mathrm{and}\;M_{01}=\sum_{u}\sum_{v}vI\left(u,v\right)$. *u* and *v* represent the row and column indices of pixels within the cropped object region, and I(u,v)∈{0,1} denotes the binary mask intensity at location (u,v).
**Algorithm 1.** Geometry-based Grasp Pose Correction
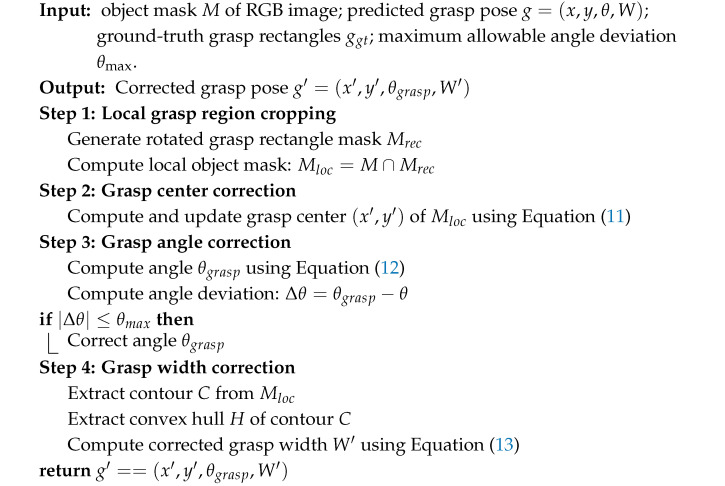


Now, we proceed to correct the angle by fitting the minimum-area bounding rectangle to the cropped object region (see Figure 5d). Let $\theta_{\mathrm{box}}$ denote the orientation of this rectangle, defined as the angle between its long side and the image *X*-axis. The grasp angle is then updated as

(12) $$\theta_{grasp}=\theta_{\mathrm{box}}+\frac{\pi }{2}.$$

To avoid the excessive grasp rotation caused by unstable long–short side assignment of the minimum-area bounding box, the grasp angle correction is constrained in this work, and no correction is applied when the angle difference exceeds $\theta_{\max}$, which is set to $30^{\circ }$.

Width correction is then performed using the convex hull (see Figure 5f). The object contour is extracted from the binary mask, and the convex hull polygon is generated from these contour points. A line passing through the corrected center along the updated grasp angle (see Figure 5g) is defined as the grasp axis. Notably, this axis is not intended to represent the object’s principal axis, but it is directly defined by the corrected grasp angle derived from the minimum-area bounding rectangle. This design avoids reliance on the potentially ambiguous principal axis estimation and ensures stable grasp pose correction for objects with weak or ill-defined geometric axes. The intersection points between this axis and the convex hull (see Figure 5h) are identified, and the distance between these points determines the corrected gripper width:

(13) $$W^{\prime }=\sqrt{{\left(x_{1}-x_{2}\right)}^{2}+{\left(y_{1}-y_{2}\right)}^{2}}+\delta ,$$

where δ is introduced to account for the inconsistency in grasp width annotations across datasets. Specifically, δ is set to 25 pixels for the Cornell dataset and 5 pixels for the Jacquard dataset in this work. By adjusting the grasp center, angle, and width, the final grasp pose (see Figure 5i) more accurately matches the object shape and decreases the risk of collisions during robotic execution.

#### 3.3.3. Dataset Verification

**Figure 6:**
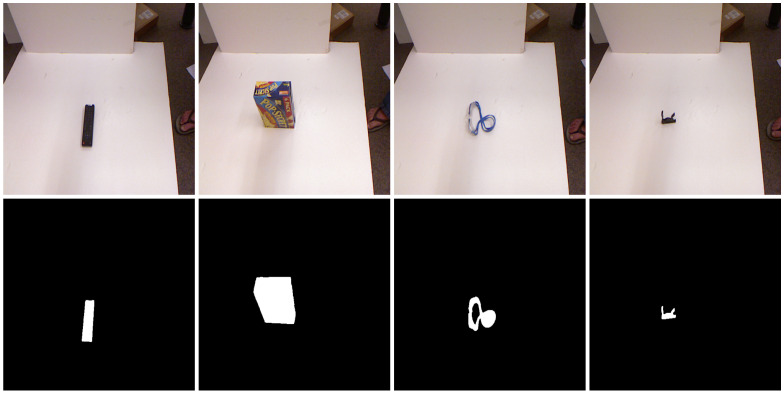
Examples for segmentation masks for Cornell dataset.

The proposed grasping algorithm is evaluated on the Cornell and Jacquard datasets [12,48]. The Jacquard dataset provides object masks, which are directly used for validation. In contrast, the Cornell dataset does not include object masks. Therefore, we generate binary masks from the RGB images using MobileSAMv2, as illustrated in Figure 6. Additionally, the Cornell dataset lacks depth images. We generate depth images from the 3D point clouds provided in the Cornell dataset by computing the Euclidean distance from each point to the camera center and projecting the result onto the corresponding RGB pixels. The resulting depth images are then combined with the RGB images to form RGB-D inputs for grasp pose prediction.

The predicted grasp pose is considered accurate if it satisfies the rectangle metric with respect to any of the annotated ground-truth grasps for the same object: the rotation angle between the ground-truth and predicted poses is within 30 degrees, and the intersection over union (IoU) score exceeds 25%. This rectangle metric, widely adopted in planar grasp benchmarks such as the Cornell and Jacquard datasets, has been extensively validated in prior studies [13,49] and enables consistent comparison with the existing methods. The accuracies of the proposed algorithm, including the versions without and with the geometric correction, are compared to those of the original GR-ConvNetv2 and four other methods in Table 2. On the Jacquard dataset, the corrected system outperforms the other methods in terms of accuracy. For the Cornell dataset, our method achieves an accuracy of 98.8%, similar to the performance of the original GR-ConvNetv2. The ablation study shows that the geometric correction maintains 98.8% accuracy on the Cornell dataset while improving the performance on the Jacquard dataset from 95.1% to 95.8%.

**Figure 7:**
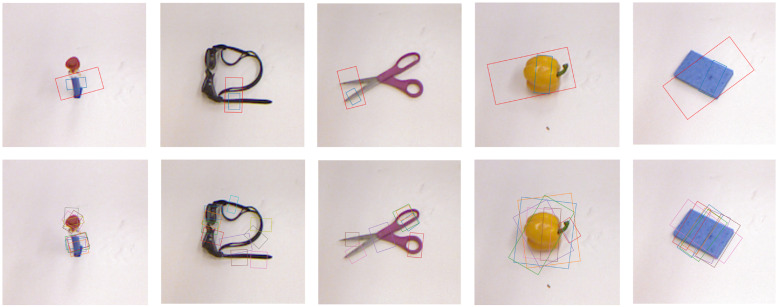
Grasp pose comparison on the Cornell dataset. The first row shows predicted (red) and corrected (blue) grasp poses, and the second row presents the corresponding ground-truth poses (shown in different colors).

**Figure 8:**
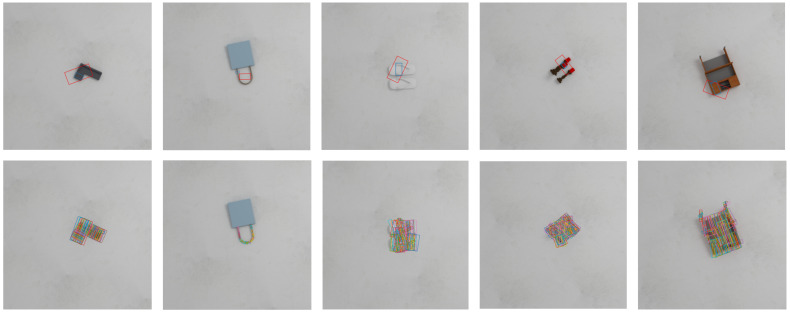
Grasp pose comparison on the Jacquard dataset. The first row shows predicted (red) and corrected (blue) grasp poses, and the second row presents the corresponding ground-truth poses (shown in different colors).

We further compare the grasp poses on the Cornell and Jacquard datasets, as illustrated in Figure 7 and Figure 8. In each figure, the first row shows the grasp rectangles predicted by the original GR-ConvNetv2 (red) and by our method (blue), while the second row shows the corresponding ground-truth poses. These figures demonstrate how our method refines the initial predictions. Specifically, the segmentation-guided masking ensures that the predicted grasp center lies on the object. The subsequent geometry-based correction adjusts the grasp center, angle, and width, resulting in corrected poses that better align with object contours and correspond to one of the ground-truth grasps in both datasets.

## 4. Experimental Studies

This section aims to provide a comprehensive experimental validation of the proposed system from both algorithmic and system-level perspectives. To this end, we first describe the hardware setup, dataset construction, and training configurations used for object detection and grasping experiments. The effectiveness of the pruned YOLOv8 detector is then evaluated in terms of the detection accuracy, robustness, and inference efficiency under different pruning ratios. Subsequently, extensive real-world grasping experiments are conducted in single-object, multi-object cluttered, and multi-object stacked scenarios under both click-based and category-specified interaction modes. Finally, the real-time performance is analyzed to demonstrate that the proposed system achieves reliable target-oriented grasping and is suitable for deployment on resource-constrained robotic platforms.

### 4.1. Experimental Setup

**Figure 9:**
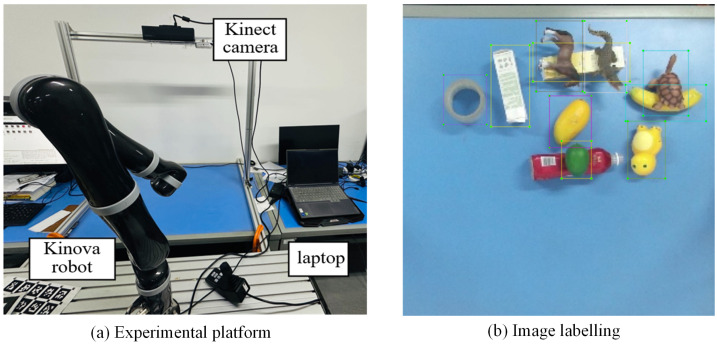
Experimental setup and annotations.

The target-oriented robotic grasping system has been implemented on a Kinova robot equipped with a two-finger gripper and a Kinect RGB-D camera, as shown in Figure 9a. A laptop with an Intel i7-9700 CPU, 16 GB of RAM, and an NVIDIA RTX 3050 GPU served as the controller of the system. Communication among the controller, the RGB-D camera, the Kinova robot was established through ROS. Before conducting the target-oriented grasping experiments, a hand-eye calibration procedure was performed to determine the transformation between the camera frame and the robot base. 

To train the YOLOv8 detection model, a dataset was constructed, consisting of 1002 RGB images captured in stacked and cluttered scenes, resulting in 4615 annotated object instances. The dataset contains ten object categories, primarily consisting of plastic toys such as a horse, crocodile, turtle, and a dinosaur, as well as fruits including a banana, mango, and a lemon, in addition to tape, bottles, and boxes. As shown in Figure 9b, all images were resized to 300 × 300 pixels and labeled using the labelImg tool. In the category-specified mode, when multiple instances of the same category are detected, the instance with the highest confidence score is selected as the grasping target, enabling efficient disambiguation without additional user interaction.

### 4.2. YOLOv8 Training and Pruning

**Figure 10:**
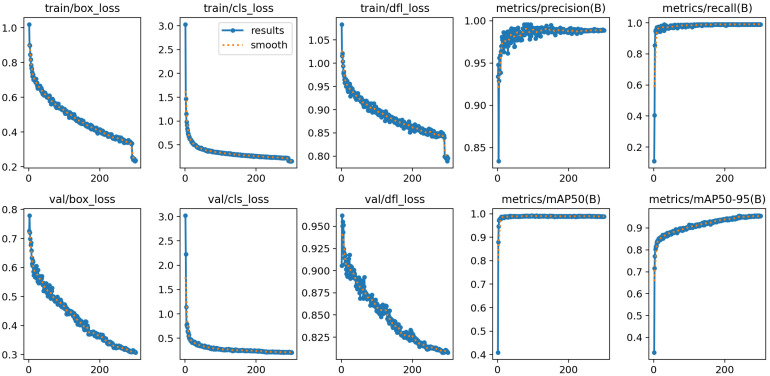
Training convergence of the YOLOv8 model.

In this work, YOLOv8 model was trained using the SGD optimizer for 300 epochs with a batch size of 16 and an initial learning rate of 0.001. The dataset was randomly divided into training, validation, and testing sets in proportions of 70%, 10%, and 20%, respectively. As shown in Figure 10, the training and validation losses decreased steadily and stabilized after approximately 200 epochs, with the model achieving high validation accuracy, indicating reliable detection and classification performance.

**Figure 11:**
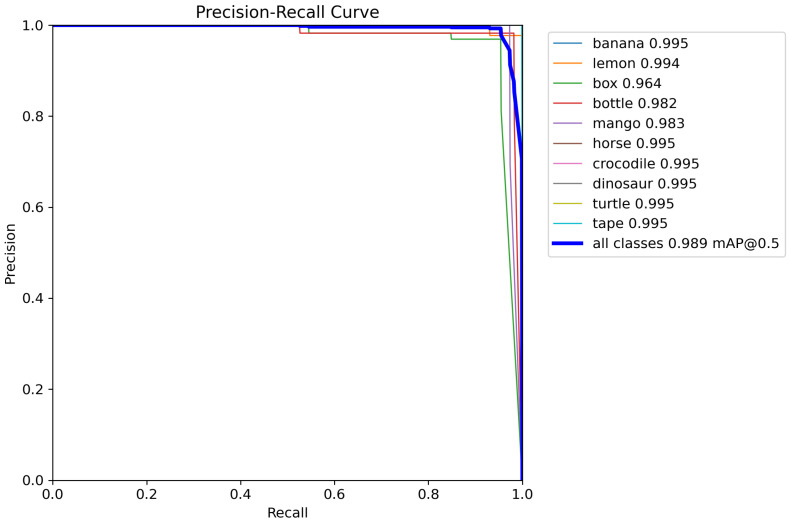
PR curve.

The detection performance was further evaluated using precision–recall (P-R) curves, as shown in Figure 11, which plot recall on the horizontal axis and precision on the vertical axis. Recall and precision are defined as Recall = TP/(TP + FN) and Precision = TP/(TP + FP), respectively. The area under each P-R curve provides the average precision (AP) for the corresponding category. The mean average precision is computed as

(14) $$\mathrm{mAP}=\frac{1}{N}\sum_{i=1}^{N}AP_{i},$$

where *N* is the number of categories. mAP_50_ denotes the AP at an IoU of 0.5, while mAP_50–95_ represents the mean across IoU thresholds from 0.5 to 0.95. Figure 11 shows that the P-R curves for all categories yield mAP_50_ values around 0.98–0.995, indicating uniformly strong detection performance across classes. The quantitative results summarized in Table 3 further validate the effectiveness of the model, where the pruned YOLOv8 detector achieves an mAP_50_ of 98.9% and an mAP_50–95_ of 95.7% on the test set.

**Figure 12:**
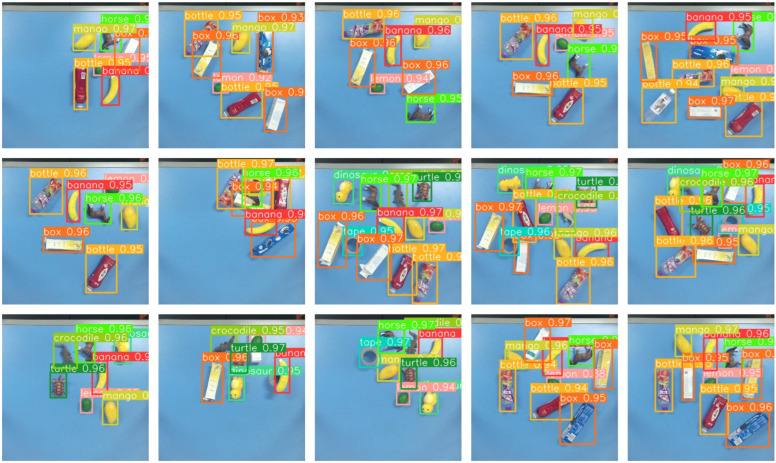
Prediction results of YOLOv8 on our dataset.

The detection results are shown in Figure 12, where the model maintains high accuracy even in cluttered and stacked scenes, demonstrating robustness for practical robotic applications.

To facilitate deployment on hardware-constrained platforms, an iterative structured pruning and fine-tuning strategy is applied to the pretrained YOLOv8 model, as described in Algorithm 2. Let $M_{0}$ denote the initial pretrained model and D the training and evaluation dataset. The objective of the proposed procedure is to progressively reduce the computational complexity of the model, measured in terms of multiply–accumulate operations (MACs), while constraining the degradation in detection accuracy.

Prior to pruning, the baseline detection performance ${\mathrm{mAP}}_{0}$ and computational cost ${\mathrm{MAC}}_{0}$ are evaluated and recorded as reference values for subsequent iterations. To ensure that the cumulative pruning ratio reaches the desired target after *K* iterations, a constant per-iteration pruning ratio *r* is computed as $r=1-{\left(1-r_{\mathrm{target}}\right)}^{\frac{1}{K}}$, which guarantees that the overall pruning effect is evenly distributed across iterations and avoids excessive structural damage in early stages. In this work, the target global pruning rate is set to $r_{\mathrm{target}}=0.5$.

The pruning procedure is performed iteratively for k=1,…,K, where K=16. At the beginning of each iteration, layers associated with the detection head are excluded from pruning to prevent severe performance degradation. This exclusion is represented by the ignored layer set $L_{\mathrm{ignore}}$, which ensures that pruning is applied only to the backbone and neck components responsible for feature extraction and fusion. Given the model from the previous iteration $M_{k-1}$, structured pruning is conducted using the operator P(·), resulting in a pruned intermediate model $M_{k}^{\mathrm{pruned}}$. In practice, P(·) is implemented using the *GroupNormPruner* from the *torch_pruning* library, implemented under PyTorch 2.1.0 framework, where channel importance is evaluated based on GroupNorm statistics with an $L_{2}$ criterion, as defined in Equation (5).
**Algorithm 2.** Iterative Structured Pruning and Fine-tuning of YOLOv8
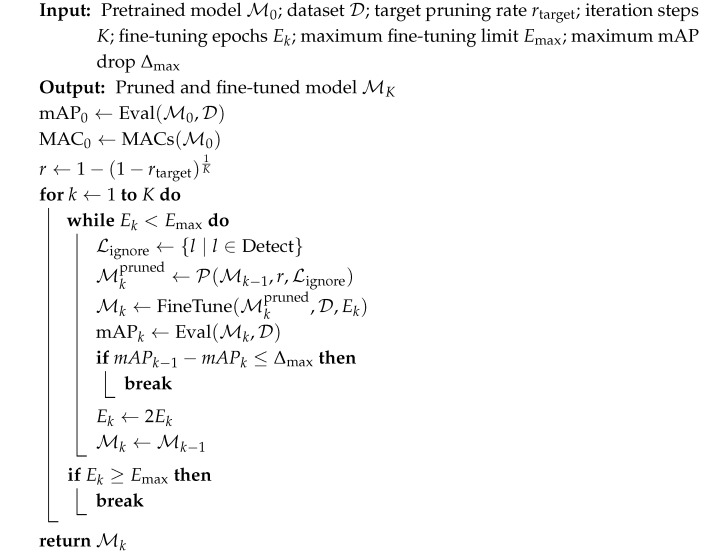


After each pruning step, the intermediate model is first evaluated as ${\mathrm{mAP}}_{k}^{\mathrm{pruned}}$ to quantify the immediate accuracy degradation caused by structured channel removal, while its computational complexity is measured as ${\mathrm{MAC}}_{k}$ to assess the achieved efficiency gain. To recover the potential performance loss, the pruned model is subsequently fine-tuned on dataset D for $E_{k}$ epochs, yielding the updated model $M_{k}$. In this work, $E_{k}$ is initially set to 50. The fine-tuning detection accuracy ${\mathrm{mAP}}_{k}$ is then compared with the baseline performance to determine whether the current pruning step is acceptable.

An early stopping and recovery criterion is introduced to prevent excessive degradation in detection accuracy during pruning. Specifically, after each pruning and fine-tuning step, the performance drop is evaluated. If the accuracy degradation satisfies ${\mathrm{mAP}}_{k-1}-{\mathrm{mAP}}_{k}\leq \Delta_{\max}$, the step is considered successful, and the procedure proceeds to the next pruning iteration. In this work, $\Delta_{\max}$ is set to 0.2. Otherwise, the algorithm reverts to the last acceptable model and increases the number of fine-tuning epochs by a factor of two, after which the pruning step is retried with the updated $E_{k}$. This recovery process continues until the accuracy constraint is satisfied or a maximum fine-tuning limit is reached (i.e., $E_{max}\geq 200$). If the limit is reached without restoring the accuracy within the predefined tolerance, the pruning process is terminated, and the previous successful model is returned. Through this iterative loop, the network is gradually compressed while maintaining the detection accuracy within a predefined tolerance. After completing all *K* iterations or triggering early termination, the final pruned and fine-tuned model is obtained for subsequent deployment and evaluation.

The detailed procedure of the pruning experiments is presented in Table 4. The baseline model at 0% pruning achieves an mAP_50_ of 98.9% with a processing speed of 93 FPS. At pruning ratios of 50% and 75%, 50 fine-tuning iterations are sufficient to restore the accuracy, and the performance drop remains within the acceptable range, with the FPS increasing to 125 and 148, respectively. At an 80% pruning ratio, 50 iterations fail to recover the accuracy, and the mAP_50_ drops to 90.9%, activating the rollback mechanism. Doubling the fine-tuning iterations to 100 restores the performance to 96.1%. At this stage, the number of parameters decreases from 3.01 M to 0.39 M, with FPS reaching 159, indicating substantial model compression with recoverable accuracy. At an 85% pruning ratio, neither 100 nor 200 fine-tuning iterations restore acceptable accuracy, and the pruning attempt is terminated. The pruned model at an 80% pruning ratio was therefore chosen as the final model, meeting the lightweight deployment requirements of the proposed system. Meanwhile, the estimated GPU memory usage decreases significantly from 0.86 GB for the baseline to 0.39 GB for the selected pruned model, demonstrating the memory efficiency gained through pruning.

**Figure 13:**
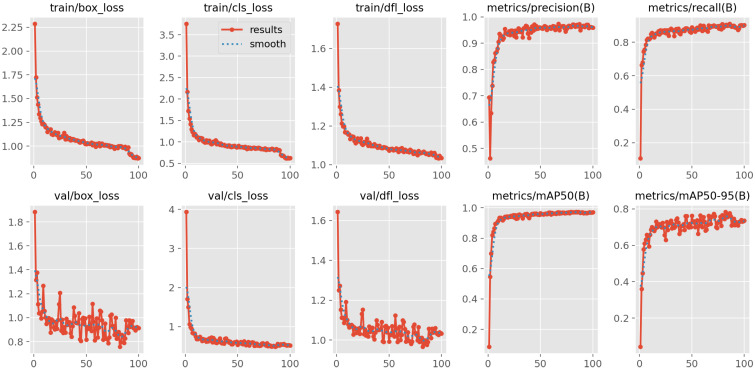
Training convergence of the YOLOv8 model pruned at 80%.

Figure 13 presents the convergence curves of the YOLOv8 model pruned at 80%, corresponding to experiment 4 in Table 4. Although slight fluctuations can be observed compared with the convergence curve of the original model shown in Figure 10, the loss decreases rapidly within the first 20 iterations, and the accuracy remains stable at a high level. These results indicate that the pruned model maintains robust learning capability and effectively captures the dataset’s information even under substantial parameter reduction.

### 4.3. Target-Oriented Grasping Evaluation

**Figure 14:**
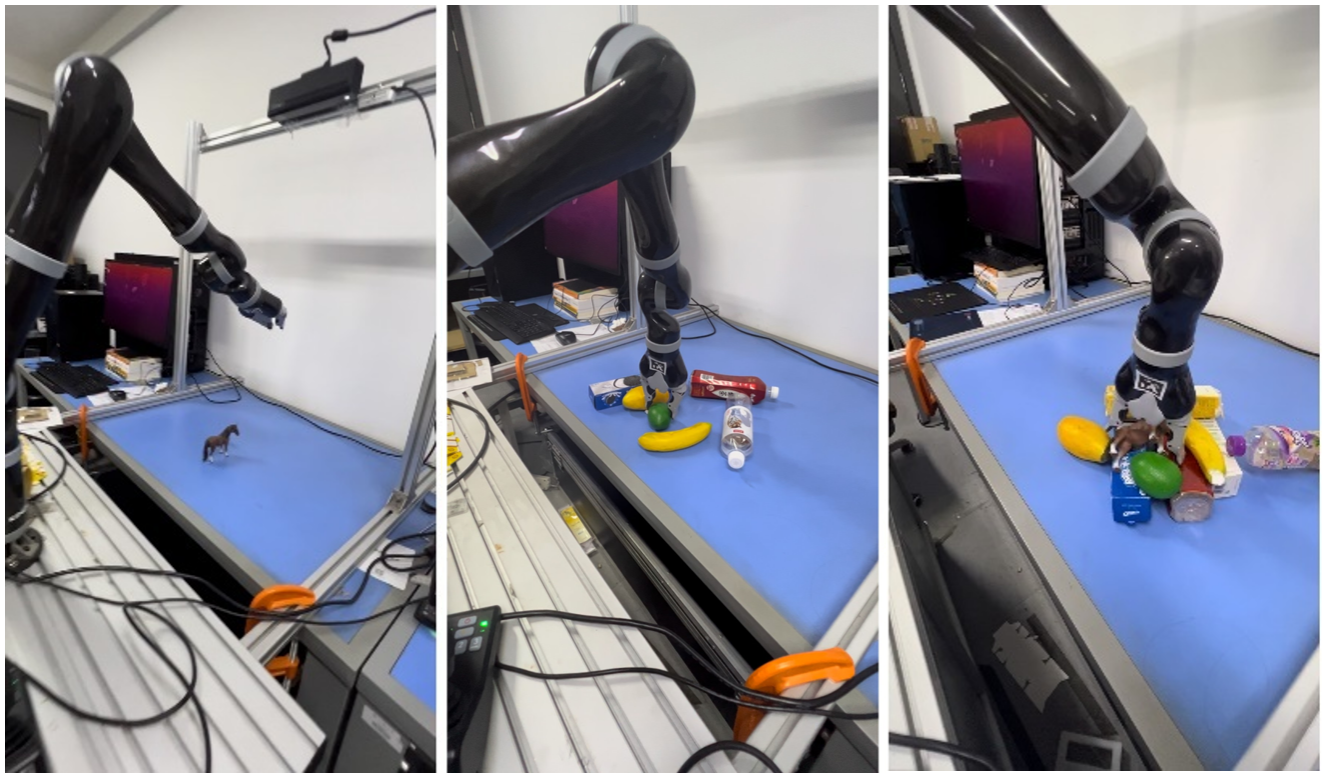
Single-object, multi-object cluttered, and multi-object stacked scenarios (from left to right).

As shown in Figure 14, the experiment evaluates 10 object categories from Section 4.1 in three scenarios: single-object, multi-object cluttered, and multi-object stacked scenarios, in the click-based mode and the category-specified mode, respectively. The click-based mode can be considered as grasping novel objects because the YOLOv8 model is not employed. A grasp is considered successful if the target object is picked up and placed at the designated location. Specifically, success requires that the object be fully lifted and stably held in the gripper without slippage or instability for two seconds. Trials involving undetected grasp rectangles, partial lifting, or unstable or slipping grasps are recorded as failures.

**Figure 15:**
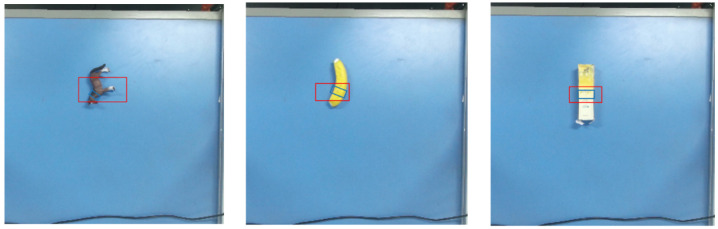
Grasp pose comparison in single-object scenario (red: GR-ConvNetv2; blue: ours).

In the single-object grasping experiments, 50 trials were conducted for each object category under each grasping mode, across 10 object categories. Figure 15 presents the visualizations of the grasp poses, where the red rectangles denote the original predictions from GR-ConvNetv2 and the blue rectangles indicate the corrected poses produced by the proposed method. It can be found that the corrected poses exhibit improved alignment with the object contours to achieve more accurate grasps.

The quantitative results are summarized in Table 5. In the click-based mode, the system achieved an average grasp success rate of 95.0%, with most object categories exceeding 94%, demonstrating stable performance even without prior object knowledge. To isolate the effect of grasp pose correction from object detection accuracy, experiments comparing the grasp success rates before and after correction were conducted exclusively in the click-based mode. The results show that the geometric correction improves the grasp accuracy. In the category-specified mode, the pruned YOLOv8 detector achieved a high overall detection accuracy of 98.8%, ensuring the reliable localization of known objects. Based on the successfully detected objects, the grasping module attained an average success rate of 94.7%, comparable to the click-based setting. Categories such as banana, lemon, turtle, and dinosaur consistently maintained high success rates, indicating that the proposed framework performs robustly across diverse object types.

Performance variations among different categories were primarily influenced by the object geometry and material properties. The mango category achieved a slightly lower success rate of 92% because its smooth and curved surface reduced friction at the contact points, causing slippage to be more likely during grasp execution. The horse category showed the lowest success rates, with 92% in the click-based mode and 89.8% in the category-specified mode, since its irregular shape and uneven mass distribution often resulted in off-center contacts and reduced grasp stability. Objects with more regular shapes and clear surface boundaries, such as the box, tape, and banana, achieved relatively higher success rates, indicating that these geometric properties allow the proposed grasp correction strategy to operate with greater stability while maintaining strong performance across diverse object categories.

**Figure 16:**
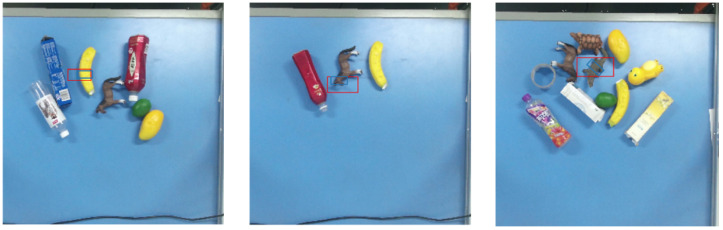
Grasp pose comparison in multi-object cluttered scenario (red: GR-ConvNetv2; blue: ours).

In the multi-object cluttered grasping experiments, objects from the 10 categories were randomly selected and arranged within the workspace in each trial, and a total of 50 attempts were conducted for each category under both modes. Figure 16 presents example grasps in cluttered multi-object settings, to demonstrate the applicability of the proposed method to more complex arrangements. Again, the corrected poses, shown as blue rectangles, exhibit improved alignment with the object contours, which can facilitate more reliable grasps and reduce collisions with surrounding objects during execution.

The results of the multi-object cluttered grasping experiments are summarized in Table 6. The click-based mode achieved an average grasp success rate of 92.2%, with most objects attaining success rates between 92% and 94%. To evaluate the impact of the geometric correction module, the grasp success rates were compared between the initially predicted poses and the corrected poses. The results indicate that grasp pose correction algorithm improved the average success rate from 88.4% to 92.2%. The category-specified mode reached an average detection accuracy of 96.4% and an average grasp success rate of 91.7%. Compared with the single-object scenario, the slightly lower performance reflects the increased difficulty in cluttered arrangements, where objects are placed close to each other and may partially occlude one another.

Similar to the single-object experiments, the mango and horse categories showed relatively lower success rates, primarily due to their curved or irregular shapes and material properties. Additionally, the crocodile category achieved a slightly reduced success rate because its irregular geometry, including protruding limbs and a long tail, increased the likelihood of interference from neighboring objects in the cluttered scenes. Objects with simpler geometries, such as the box, tape, and banana, were less affected by neighboring objects and consistently achieved high success rate.

**Figure 17:**
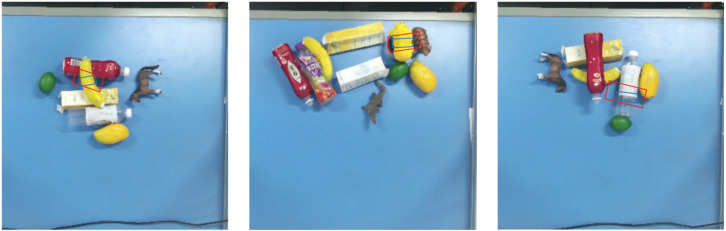
Grasp pose comparison in multi-object stacked scenario (red: GR-ConvNetv2; blue: ours).

In the multi-object stacked grasping experiments, objects from the 10 categories were also randomly selected and placed in vertically overlapped configurations, and 50 grasping attempts were conducted for each category under both modes. The corrected grasp poses are displayed in Figure 17, which demonstrates the grasp accuracy improvement obtained from the proposed method for the multi-object stacked scenario.

As shown in Table 7, the click-based mode achieved a grasp success rate of 89.6%, while the category-specified mode reached a detection accuracy of 92.2% and a grasp success rate of 89.2%. Compared with the single-object and cluttered scenarios, the grasping performance exhibits a moderate decline, primarily due to the stacked setting of objects. The stacking can lead to significant vertical overlap and mutual occlusion, which increase the sensitivity of the gripper’s descent height and make collisions with neighboring objects more likely. Specifically, in the click-based mode, grasping using the initially predicted poses resulted in a lower success rate of 85%, mainly because the predicted grasp width were sometimes too wide and the poses insufficiently accurate, leading to collisions. After applying the geometric correction algorithm, the success rate improved significantly to 89.6%.

To evaluate the real-time performance of the proposed framework, all experiments were conducted on a consumer-level laptop as described in Section 4.1. Table 8 reports the inference time of each module under the two user interaction modes, measured separately on both GPU and CPU configurations. In the click-based mode, the total processing time per frame is 67 ms on the GPU, whereas in the category-specified mode, it increases to 75 ms, due to the additional YOLOv8 object detection step. In CPU mode, the total processing time increases significantly, with the click-based mode taking 407 ms per frame and the category-specified mode taking 499 ms. The memory usage also rises to approximately 7.5 GB, compared to 2 GB in GPU mode. This increase is mainly due to the fact that all model parameters and intermediate feature maps are stored and computed in system RAM during CPU inference, leading to a higher memory demand for GR-ConvNetv2 pose prediction, YOLOv8 detection, and MobileSAMv2 segmentation. These results demonstrate that the proposed framework can achieve target-oriented grasping in real time and is suitable for deployment on mobile robotic platforms, especially when GPU acceleration is available.

## 5. Discussion

The proposed lightweight target-oriented grasping framework is designed to enable accurate and efficient target-oriented grasping on resource-constrained robotic platforms. The framework combines a pruned YOLOv8 detector for efficient object localization, MobileSAMv2 to generate object-specific masks that constrain candidate grasp regions, and a geometry-based correction module that refines the grasp pose, angle, and width. Together, these elements enable precise and stable grasping in single-object, cluttered, and stacked scenarios, supporting the interactive grasping of known or novel objects.

To enable efficient target-oriented grasping on resource-constrained robotic platforms, YOLOv8 was compressed from 3.01 M to 0.39 M parameters using a structured pruning strategy, increasing the inference speed from 93 FPS to 159 FPS. It should be noted that YOLOv8 was trained on a relatively small custom dataset consisting of ten household objects, which may limit its generalization to unseen objects. Future work will focus on leveraging larger and more diverse datasets to enhance robustness. For grasp pose prediction, the framework uses the efficient pretrained GR-ConvNetv2 for fast and accurate estimation. Unlike LLMs or computation-heavy detection and segmentation methods that require high-performance hardware, the proposed framework can be deployed in real time on consumer-level devices such as a laptop with an Intel i7-9700 CPU and RTX 3050 GPU for interactive grasping of user-specified objects.

MobileSAMv2 generates segmentation masks for the user-specified target object, constraining the highest-quality grasp candidates to the object region and suppressing interference from background objects. MobileSAMv2 is trained on large-scale datasets and generally provides accurate segmentation results, even in cluttered or stacked scenes. Occasional boundary inaccuracies, such as slight over- or under-segmentation, have limited impact on grasp pose prediction, since GR-ConvNetv2 assigns higher quality scores to grasp candidates concentrated near the object center rather than along object edges. As a result, the highest-quality grasp candidates typically remain within the correctly segmented central region. During geometry-based pose correction, such boundary deviations may introduce minor errors in the estimation of the object center or grasp width, but these effects are negligible unless segmentation errors are substantial, leading to robust grasp success rates in cluttered and stacked scenarios.

Furthermore, the geometry-based grasp correction relies on contour-derived geometric cues to refine the predicted grasp pose. While this strategy is effective in most practical scenarios, its behavior can be influenced by the local contour characteristics within the grasp region. In particular, for objects with highly irregular or strongly non-convex shapes, local geometric variations may affect the orientation estimation and introduce a bias in the corrected grasp pose, which can be reflected in the grasp IoU metric. It is important to note that such cases mainly arise for objects with extreme shape irregularities and occur infrequently in typical scenes. Overall, the proposed correction module remains robust for common object geometries, while these observations highlight potential directions for further improving robustness in handling complex contours. Beyond object shape characteristics, the overall system also remains robust under varying lighting conditions and sensor noise. Although the depth noise may slightly affect the initial RGB-D-based grasp prediction, the subsequent pose correction effectively compensates for these discrepancies. Since the correction relies on binary segmentation images rather than RGB inputs, the estimation of the grasp center, angle, and width is largely independent of lighting variations and sensor noise.

Across all experiments, no cases were observed in which the corrected grasp width exceeded the physical limits of the gripper. This is because the grasp widths are constrained by both the object geometry and the gripper specifications. The evaluated objects are compatible with the Kinova gripper’s maximum opening (approximately 17 cm), and the proposed geometry-based correction further bounds the final grasp width using the object’s convex hull. As a result, the corrected grasp widths remain physically feasible during execution.

Extensive experiments demonstrate the effectiveness of the proposed approach. The framework achieved grasp success rates of 98.8% on the Cornell dataset and 95.8% on the Jacquard dataset, with over 90% success in single-object and cluttered scenarios. In the category-specified mode, the object detection accuracy exceeded 92% across all three scenarios. Additionally, the framework demonstrated real-time performance on resource-constrained hardware, with the total inference time per frame being 67 ms in the click-based mode and 75 ms in the category-specified mode on the GPU.

To balance the system simplicity and real-time performance, the current framework relies on a single top-view RGB-D image, which may limit the grasping accuracy in multi-object stacked scenarios with severe occlusions along the Z direction. Future work will explore extending the framework to multi-view RGB-D inputs by incorporating depth fusion or lightweight 3D reconstruction techniques to better resolve occlusions. In addition, the integration of closed-loop visual or tactile feedback will be investigated to further enhance the grasp reliability, adaptability, and robustness under challenging conditions, such as deformable objects, complex geometries, and heavy occlusions.

## 6. Conclusions

Aiming at target-oriented grasping on resource-constrained mobile robots, we propose a lightweight and efficient framework that supports both click-based grasping for novel objects and category-based grasping for known objects. To reduce the model complexity while maintaining the detection accuracy, YOLOv8 is compressed using a structured pruning method and combined with MobileSAMv2 to generate target-specific masks that guide grasp proposals efficiently. For grasp pose generation, a pretrained GR-ConvNetv2 predicts candidate grasps, which are further refined by a geometry-based correction module that adjusts the position, angle, and width of the initial grasps to improve stability and reduce collisions. Finally, a target-oriented grasping system has been implemented on a Kinova robot, with all models deployed on a consumer-level laptop. Real-world experiments across single-object, cluttered, and stacked scenarios verify that the proposed system maintains reliable grasping performance and stable object detection, while also supporting real-time execution under both interaction modes.

Overall, the proposed framework provides a practical and robust approach for target-oriented grasping of both known and novel objects in cluttered and stacked environments. By integrating lightweight object detection, grasp pose generation, and flexible interaction modes, it achieves reliable performance in real-world tasks and is well suited for deployment on resource-constrained mobile robots in household and industrial environments.

## Figures and Tables

**Table 1:** Summary of representative target-oriented grasping methods.

Reference	Work Category	Applied Methods	Key Strengths	Limitations	Inference Time	Hardware
[30]	Detection-and-grasp	Multi-task DSSD for object detection, semantic segmentation, and grasp detection	The method jointly performs object detection, semantic segmentation, and grasp detection in a single-shot framework, achieving higher accuracy and lower computational cost than separate models.	The method relies on depth information for grasp execution, leading to reduced success rates when depth sensing is unreliable.	None	None
[31]	Detection-and-grasp	YOLOv8-BFS for object detection, Gen6D-Op for 6-DoF pose estimation	The framework achieves high-precision detection, pose localization, and real-world grasping of chemical vials in laboratory scenarios.	The performance depends on the depth accuracy and remains limited for transparent, liquid-filled, and non-prismatic objects.	YOLOv8-BFS: 129.9 FPS	Elite EC66 six-axis arm, HITBOT Z-EFG-100 gripper, Intel RealSense D435i camera, NVIDIA RTX 4060 Laptop GPU
[32]	Detection-and-grasp	YOLO-CMA for object detection, PointNet++ for grasp estimation	The system enables robust 6-DoF and collision-free grasping in cluttered multi-object scenes through objective-oriented grasp planning.	The system lacks adaptive replanning and fails when the target object is partially or fully occluded.	None	Intel i9-11900K CPU, NVIDIA RTX 4090 GPU, UR3 robot, Robotiq-2F gripper, RealSense D435 camera
[29]	Detection-and-grasp	YOLOv11, FAST-SAM for object detection; PCA-based pose estimation; centroid-based 6-DoF grasp estimation	The system provides a lightweight and energy-efficient solution for grocery pick-and-pack tasks with robust performance on edge devices.	The PCA-based pose estimation is sensitive to object geometry and may misclassify near-symmetric objects.	Under 5 s per item	UR16e robot, MiR100 platform, ZED2i stereo camera, Jetson Orin Nano computer
[18]	Detection-and-grasp	YOLOv3 for object detection, GG-CNN for grasp estimation	The method combines fast object detection and grasp prediction, achieving high speed and accuracy in real-world robotic experiments.	The method is limited to object categories seen during YOLO training and cannot generalize to unknown objects.	35.7 FPS	6-DOF KINOVA robot, Intel i7-11700H CPU, RealSense D435i camera
[19]	LLM-based grasping	Pretrained LLaVA with LoRA fine-tuning for target identification, GR-ConvNet for grasp prediction	The framework enables reasoning-based grasping from implicit natural language instructions and demonstrates strong performance in cluttered scenes.	The framework shows limited capability in grasping novel objects and refining grasp poses through conversational interaction.	None	None
[5]	LLM-based grasping	GraspGPT with SAM-based object segmentation and Contact-GraspNet for grasp candidate generation	The method leverages LLM-based semantic knowledge to enable task-oriented grasping with zero-shot generalization to novel objects.	The method relies on single-view point clouds and external grasp candidate generation, limiting robustness in complex scenarios.	None	Desktop PC with NVIDIA RTX 3090 GPU, Kinova Gen3 7-DoF robot, parallel gripper, RealSense D435 camera
[37]	VLA-based grasping	DexGraspVLA with hierarchical planner–controller architecture	The framework supports long-horizon tasks, non-prehensile grasping, and robustness to disturbances through foundation models.	The framework does not address functional grasping or incorporate tactile feedback for manipulation refinement.	None	RealMan RM75-6F robot, PsiBot G0-R hand, RealSense D435 cameras, 8×A800 GPU server or online APIs
[36]	VLA-based grasping	VLA-Grasp with multimodal encoders and cross-attention fusion	The framework integrates vision, language, and action to support multi-grasp decision-making and task-oriented grasping.	The reliance on online LLM querying introduces significant delays when encountering new objects or tasks.	1.91 s	Desktop server with dual RTX 3090Ti GPUs, 6-DOF robotic arm, two-finger gripper, Orbbec Femto-W camera
[33]	VLM-based grasping	CLIP-driven Attribute-aware Network (CTNet)	The method enables efficient and robust language-conditioned visual segmentation and grasping through attribute-aware multimodal interaction.	The method does not consider physical object properties, which may lead to gripper collision or slippage.	26.7 ms	UR3e robot, Robotiq 140 gripper, RealSense D435 camera, two RTX 3090 GPUs
[20]	VLM-based grasping	CLIP-Adapter combined with GraspNet (OVGrasp)	The framework enables open-vocabulary grasping of unseen object categories in cluttered environments without predefined labels.	The framework struggles with delicate or irregular objects and is sensitive to lighting conditions and transparency.	None	UR5 robot, DH-ROBOTICS AG-95 gripper, RealSense D435i camera

**Table 2:** Accuracy comparison on Cornell and Jacquard datasets.

Authors	Algorithms	Cornell Accuracy (%)	Jaquard Accuracy (%)
Wang et al. [28]	TF-Grasp	97.99	94.6
Liu et al. [50]	Mask-RCNN + Y-Net + Q-Net	95.2	92.1
Zhou et al. [51]	DSC-GraspNet	98.3	94.7
Kumra et al. [27]	GR-ConvNetv2	98.8	95.1
Mei et al. [52]	RoG-SAM-GT	98.8	94.6
Ours	GR-ConvNetv2 + MobileSAMv2	98.8	95.1
Ours	GR-ConvNetv2 + MobileSAMv2 + Correction	98.8	95.8

**Table 3:** Test results on our dataset.

Category	Precision (%)	Recall (%)	mAP_50_ (%)	mAP_50–95_ (%)
Banana	97.7	99.8	99.5	97.7
Lemon	97.6	99.3	99.4	95.6
Box	96.8	95.5	96.4	94.1
Bottle	98.2	98.2	98.2	98.2
Mango	99.5	97.3	98.3	95.9
Horse	99.8	99.4	99.5	97.0
Crocodile	99.7	99.5	99.5	93.9
Dinosaur	99.6	99.8	99.5	93.6
Turtle	99.5	99.6	99.5	94.9
Tape	99.8	99.7	99.5	97.0
All	98.8	98.8	98.9	95.7

**Table 4:** YOLOv8 pruning experiment with estimated GPU memory usage.

Experiments	Pruning Ratio (%)	Fine-Tuning Iterations	Parameters	mAP50 (%)	Results	Inference Speed (FPS)	GPU Memory (GB)
0	0	0	3,007,598	98.9	Baseline	93	0.86
1	50	50	920,028	98.9	Success	125	0.63
2	75	50	448,109	97.3	Success	148	0.48
3	80	50	385,413	90.9	Failure	/	/
4	80	100	385,413	96.1	Success	159	0.39
5	85	100	330,481	65.0	Failure	/	/
6	85	200	330,481	55.4	Failure	/	/

**Table 5:** Results of single-object grasping experiment.

	Click-Based Grasping				Category-Specified Grasping			
Category	Predicted Num. of Grasping (Succ./Total)	Predicted Success Rate (%)	Corrected Num. of Grasping (Succ./Total)	Corrected Success Rate (%)	Num. of Detection (Succ./Total)	Accuracy (%)	Num. of Grasping (Succ./Total)	Success Rate (%)
Banana	49/50	98	49/50	98	50/50	100	49/50	98
Lemon	47/50	94	48/50	96	50/50	100	48/50	96
Box	48/50	96	48/50	96	49/50	98	47/49	95.9
Bottle	46/50	92	47/50	94	48/50	96	45/48	93.7
Mango	45/50	90	46/50	92	50/50	100	46/50	92
Horse	44/50	88	46/50	92	49/50	98	44/49	89.8
Crocodile	45/50	90	47/50	94	50/50	100	46/50	92
Dinosaur	47/50	94	48/50	96	50/50	100	49/50	98
Turtle	46/50	92	48/50	96	50/50	100	48/50	96
Tape	47/50	94	48/50	96	48/50	96	46/48	95.8
All	464/500	92.8	475/500	95.0	494/500	98.8	468/494	94.7

**Table 6:** Results of multi-object cluttered grasping experiment.

	Click-Based Grasping				Category-Specified Grasping			
Category	Predicted Num. of Grasping (Succ./Total)	Predicted Success Rate (%)	Corrected Num. of Grasping (Succ./Total)	Corrected Success Rate (%)	Num. of Detection (Succ./Total)	Accuracy (%)	Num. of Grasping (Succ./Total)	Success Rate (%)
Banana	45/50	90	46/50	92	49/50	98	46/49	93.9
Lemon	45/50	90	47/50	94	50/50	100	47/50	94
Box	45/50	90	46/50	92	50/50	100	47/50	94
Bottle	45/50	90	47/50	94	49/50	98	45/49	91.8
Mango	43/50	86	45/50	90	50/50	100	45/50	90
Horse	41/50	82	44/50	88	47/50	94	41/47	85.1
Crocodile	42/50	84	45/50	90	48/50	96	43/48	89.6
Dinosaur	45/50	90	47/50	94	47/50	94	43/47	91.5
Turtle	45/50	90	46/50	92	47/50	94	43/47	91.5
Tape	46/50	92	48/50	96	45/50	90	42/45	93.3
All	442/500	88.4	461/500	92.2	482/500	96.4	442/482	91.7

**Table 7:** Results of multi-object stacked grasping experiment.

	Click-Based Grasping				Category-Specified Grasping			
Category	Predicted Num. of Grasping (Succ./Total)	Predicted Success Rate (%)	Corrected Num. of Grasping (Succ./Total)	Corrected Success Rate (%)	Num. of Detection (Succ./Total)	Accuracy (%)	Num. of Grasping (Succ./Total)	Success Rate (%)
Banana	44/50	86	46/50	92	46/50	92	42/46	91.3
Lemon	42/50	84	45/50	90	48/50	96	45/48	93.8
Box	44/50	88	46/50	92	47/50	94	42/47	89.4
Bottle	45/50	88	46/50	92	46/50	92	41/46	89.1
Mango	41/50	82	43/50	86	47/50	94	40/47	85.1
Horse	38/50	76	41/50	82	45/50	90	38/45	84.4
Crocodile	41/50	82	44/50	88	46/50	92	40/46	87.0
Dinosaur	43/50	84	45/50	90	46/50	92	42/46	91.3
Turtle	43/50	86	46/50	92	46/50	92	41/46	89.1
Tape	44/50	88	46/50	92	44/50	88	40/44	90.9
All	425/500	85	448/500	89.6	461/500	92.2	411/461	89.2

**Table 8:** Inference time of each module for two user interaction modes on GPU and CPU (ms).

Module	Click-Based Mode (GPU/CPU)	Category-Specified Mode (GPU/CPU)
GR-ConvNetv2 pose prediction	14/75	14/75
Pruned YOLOv8 detection	/	8/92
MobileSAMv2 segmentation	48/327	48/327
Geometry-based correction	5/5	5/5
Total	67/407	75/499

## Data Availability

Data are contained within the article.
